# Evaluation of pain and function after two home exercise programs in a clinical trial on women with chronic neck pain - with special emphasises on completers and responders

**DOI:** 10.1186/1471-2474-15-6

**Published:** 2014-01-08

**Authors:** Linn Karlsson, Esa-Pekka Takala, Björn Gerdle, Britt Larsson

**Affiliations:** 1 Rehabilitation Medicine, Department of Medicine and Health Sciences (IMH), Faculty of Health Sciences, University of Linköping, and Pain and Rehabilitation Centre, UHL, County Council SE 581 85 Linköping, Sweden; 2 Work-related Diseases, Finnish Institute of Occupational Health, Topeliuksenkatu 41 FI 00250 Helsinki, Finland

**Keywords:** Neck pain, Shoulder pain, Home exercise, Strength training, Stretching, Function

## Abstract

**Background:**

Different types of exercises can help manage chronic neck pain. Supervised exercise interventions are widely used, but these protocols require substantial resources. The aim of this trial, which focused on adherence, was to evaluate two home exercise interventions.

**Methods:**

This parallel group randomized controlled trial included 57 women randomly allocated into two groups – a strength training group (STRENGTH, 34 subjects) and a stretching group (STRETCH, 23 subjects). The interventions focused on the neck and shoulder muscles and lasted for 12 months. The STRENGTH group performed weight training and ended each session with stretching exercises. These stretching exercises constituted the entirety of the STRETCH group’s training session. Both groups were instructed to exercise three times per week. All the participants kept an exercise diary. In addition, all participants were offered support via phone and e-mail. The primary outcomes were pain intensity and function. The trial included a four- to six-month and a twelve-month follow-up. A completer in this study exercised at least 1,5 times per week during eight unbroken weeks. A responder in this study reported clinically significant improvements on pain and function. The statistical analyses used the Mann Whitney U-test, Wilcoxon signed-rank test, and *X*^*2*^ test.

**Results:**

At four- to six-months, the numbers of completers were 19 in the STRENGTH group and 17 in the STRETCH group. At twelve months, the corresponding numbers were 11 (STRENGTH) and 10 (STRETCH). At four- to six-months, the proportions of subjects reporting clinically important changes (STRENGTH and STRETCH) were for neck pain: 47% and 41%, shoulder pain: 47% and 47%, function: 37% and 29%. At twelve months, the corresponding numbers were for neck pain: 45% and 40%, shoulder pain: 55% and 50%, function: 55% and 20%.

**Conclusions:**

No differences in the two primary outcomes between the two interventions were found, a finding that may be due to the insufficient statistical power of the study. Both interventions based on home exercises improved the two primary outcomes, but the adherences were relatively low. Future studies should investigate ways to improve adherence to home exercise treatments.

**Trial registration:**

ClinicalTrials.gov Id: NCT01876680

## Background

Because chronic neck pain has a high prevalence rate (20% - 60%) with considerable socioeconomic costs [1,2], efficient and cost-effective treatments are needed. Compared to healthy controls, patients with neck pain exhibit weaker neck muscle strength [3-5]. Some research suggests that insufficient strength in the neck and shoulder muscles may cause muscle pain. Hence, with weak muscles, the relative workload in daily activities will be too high resulting in muscle pain [6]. In addition, if activities are painful, it is likely that movements and force will be inhibited [6,7], increasing the relative workload.

Different types of exercises are commonly used to treat neck and shoulder pain. Specific strength training can decrease pain [8,9], decrease disability [10], and increase strength in the neck/shoulder muscles [8,10]. The scientific knowledge about how different exercises affect pain conditions, however, is incomplete [11]. Some studies [12-19] have shown that different types of training (e.g., endurance, coordination, aerobic, and stretching) may also reduce neck/shoulder muscle pain. A recent systematic review [11] concluded that there still is no clear evidence on which exercises or dosages most effectively reduce pain and increase function in non-specific neck pain. The studies with reported results in favour of strength training include protocols for three types of supervised training: fully [8], partially [9], or initially [10]. Supervised training, however, requires specific and substantial resources and may be difficult to apply in a clinical context.

Adherence to prescribed exercise should be considered in evaluations of exercise interventions. Supervised or individualized interventions and self-management techniques may enhance exercise adherence [20]. More research, however, is needed to explore different aspects of adherence and its relation to positive clinical outcome [20,21]. To our knowledge, no studies have evaluated neck pain treatments using solely home-based exercise interventions. Moreover, we have found no studies that have reported analyses of adherence in association with the results of home-based exercise interventions.

The aim of this trial was to evaluate two home exercise interventions, one year of strength exercises and one year of stretching exercises. Within this aim, we have focused on adherence to the prescribed interventions. The participants were women with chronic neck pain who were recruited from a general population. Primary outcomes were pain intensity and function.

Based on previous studies [8,10], we anticipated that pain would decrease and function would increase at the 4- to 6-month follow-up and further improve after one year of training. We expected the results to be more pronounced in the strength-training group and that greater adherence to the exercise interventions would produce better results.

## Methods

### Trial design

Between September 2009 and February 2011, a randomized, controlled, parallel-group trial was performed at Linkoping University Hospital (Linkoping, Sweden). The study was approved by the regional ethical committee of Linköping University, diary number M10-80.

### Participants

**Figure 1:**
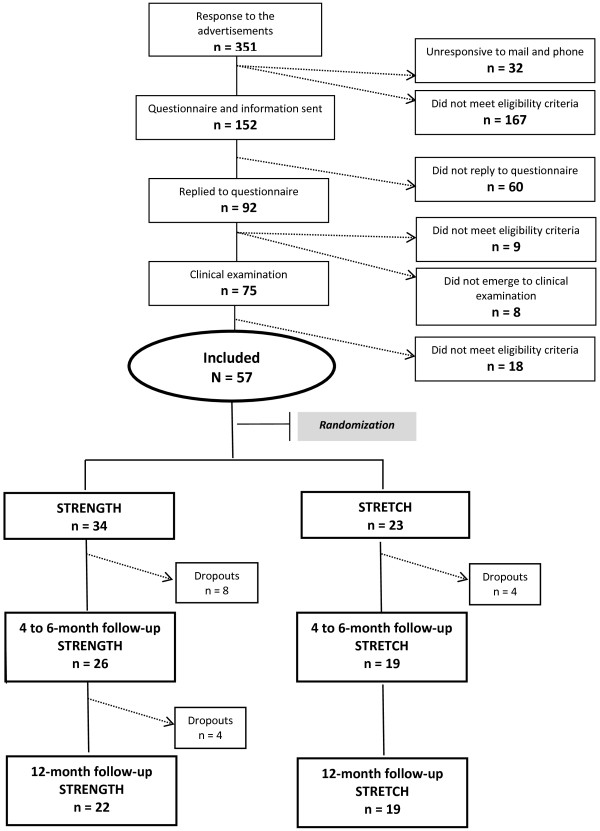
Flowchart of participants.

The participants were recruited from the general population through advertisements in local newspapers. Respondents to the advertisements were informed about the study and interviewed by phone to determine whether they met the inclusion criteria. The respondents were mailed a package that included a detailed letter about the study, the Nordic Style Questionnaire (NSQ) [22,23], and a Swedish version of the Neck Disability Index (NDI) [24]. The NSQ provided specific information on pain location and intensity for the previous 12 months and the NDI provided information about function. Before acceptance into the study, each respondent underwent a standardized clinical examination of the neck and upper extremities [25,26] performed by a physiotherapist especially trained for this task and who was blinded with respect to group affiliation. This examination included questions on pain, tiredness, and stiffness as well as physical tests and palpation that evaluated range of motion, tightness of muscles, pain sensitivity, and muscle strength. Patients having symptoms consistent with the diagnosis of tension neck syndrome – neck pain, sense of fatigue, or stiffness in the neck, pain spreading from the neck to the back of the head, tightness of muscles, and tender spots in the muscles [27] – were included in the study. All the participants (57 women) signed an informed consent before entering the study. A flowchart of participants is presented in Figure 1.

Inclusion criteria were female, 20 – 60 years old, and constantly or frequently occurring pain in the neck/shoulder area for more than six months. In addition, symptoms consistent with the clinical diagnosis of tension neck syndrome [27] was required with a pain intensity of at least 3 on the Numeric Rating Scale (NRS) [28] and/or a reduction in function scored as at least 10 (see “Neck function” for details) measured by the Swedish version of the NDI [24]. The participant also had to declare that they were motivated to follow the exercise protocol. Exclusion criteria were widespread pain, major trauma in medical history, pregnancy, inflammatory and hormonal disorders, neurological causes of the pain, tendonitis in upper extremities, and severe psychiatric illness.

### Randomization and blinding

The participants were randomly assigned to either a strength-training group (STRENGTH) or a stretching group (STRETCH). The inclusion process continued for six months and participants entered the trial in groups, which started every second week. The randomization was performed by randomly selecting the start-up sequence for the group affiliations using the computer program Minitab v. 15. (Minitab Inc., http://www.minitab.com). Participants were assigned to groups in a consecutive manner until it was time for the groups to start exercising. The two intervention groups were similar in age, duration of pain, pain intensity, and function according to the NDl (Table 1).

**Table 1:** Baseline characteristics for the strength training group (N = 34) and the stretching group (N = 23)

	**STRENGTH**	**n**	**STRETCH**	**n**	**p-value**
	**Median (25, 75 percentile)**		**Median (25, 75 percentile)**		
**Age (years)**	46 (40, 50)	34	42 (33, 47)	23	0.268
**Pain duration (years)**	10 (6, 15)	32	6 (4, 13)	22	0.053
**Pain intensity neck (NRS)**	6 (4, 7)	33	5 (4, 6)	23	0.538
**Pain intensity shoulder (NRS)**	4 (3, 6)	33	5 (3, 7)	23	0.873
**Function (NDI)**	13 (10, 18)	33	14 (11, 18)	22	0.897

NRS = Numeric Rating Scale, NDI = Neck Disability Index.

The examiner conducting the standardized clinical examination of the neck and upper extremities during the inclusion process was blinded with respect to group affiliation. There were no other components of blinding in the trial due to limited resources.

### Interventions

The exercises were modified from previous studies [8,10]. The STRENGTH group started with specific strength training that focused on the neck and shoulder muscles. The strength exercises included arm abduction, upright row, biceps curls, flys, reverse flys, and pullovers. Dumbbells were used in all these exercises. Additionally, lifting the head up (without resistance) from a supine position was also performed as a strength exercise. The strength training was progressive and periodized throughout the training period. For the first eight weeks, the participants learned to perform the exercises correctly and to adopt the principles of progressive training. During this learning period, 2-kg dumbbells were used with the goal of performing three sets of 20 repetitions for each exercise. The subjects were instructed to do as many repetitions as possible to progressively reach 20 repetitions. A majority of the subjects did not manage to do 20 repetitions until the end of the eight-week period. After eight weeks, the weight of the dumbbells was individually adjusted to the heaviest weight possible to perform ten repetitions. During the remaining training period of one year, the strength training consisted of three weeks of exercise with the heaviest weight possible (three sets of ten repetitions) and one week of exercise with 2-kg dumbbells (three sets of 20 repetitions). The subjects were instructed to progressively perform up to 15 repetitions with the heaviest weights. When they managed to do three sets of 15 repetitions, they were instructed to adjust the load again to the heaviest load that would allow them to perform ten repetitions. The exercise lifting the head up from supine were performed without resistance other than the weight of their head. First, the subjects were instructed to do as many repetitions as possible with the goal of 3 sets of 20 repetitions. After that, they were instructed to increase the number of repetitions. Three series of dynamic exercises for the trunk and legs (sit-ups, back extensions, and squats) followed the strength training. Each exercise was performed 20 times. Results on core and leg strength are not presented in this paper.

Stretching exercises for the neck, shoulders, and upper limb muscles ended the exercise session for the STRENGTH group and constituted the complete exercise session for the STRETCH group. The stretching exercises were the same as used in a previous study [10], which comprised retraction of the neck and stretching the following muscles: m. trapezius upper and middle portion, m. sternocleidomastoideus, m. rhomboids, m. pectoralis major, and the flexors and extensors of the wrist.

To learn how to conduct the prescribed home exercise program, each participant was invited to three instruction sessions. All participants were then asked to do the exercise session three times a week and they were encouraged to perform an optional aerobic exercise for 30 minutes with the same frequency. The STRENGTH group was instructed to give priority to the specific strength exercises if they could not manage to perform the complete session. The participants were encouraged to organize their home exercise so that it would fit into their everyday life. All participants used an exercise diary to record exercise frequency. The exercise diary was also supposed to help motivate the participants to exercise. The exercise diary was based on long-term and short-term goals, the latter also functioning as a detailed exercise plan. Furthermore, the exercise diary contained a weekly evaluation of the implementation of the training. The diary had the same structure for both the STRENGTH and the STRETCH group. Furthermore, support for adherence to the home exercise programme was provided by phone or e-mail every four to eight weeks. The support was more frequent at the beginning of the one-year training period and it was conducted in the same way for both intervention groups. The introduction to the home-exercise program and the support were performed by a physiotherapist experienced in this area.

### Outcomes

#### Outcome measurement time points

Both primary and secondary outcomes were measured at baseline (BL) one day about two weeks before the start of the intervention. All outcomes were followed up in the same manner after four to six months of training (4 to 6 months) and after 12 months of training (12 months). The follow-up time point in the middle of the trial (4 to 6 months) varied due to practical reasons such as on-going recruitment phase, participants summer vacations, and the fact that the same investigators performed all outcome measurements. The outcome measures were made by skilled physiotherapists.

Primary outcomes were pain intensity and neck function. Secondary outcomes were range of motion in the neck, neck strength, and shoulder strength.

#### Pain intensity

The participants assessed their pain intensity in the neck and shoulders during the previous week by marking on an 11-grade (0 – 10) Numeric Rating Scale (NRS). Zero indicated no pain at all and 10 indicated worst pain possible [28].

#### Neck function

Self-reported neck function was measured using the Swedish version of the NDI [24]. The NDI includes ten items affected by neck pain: pain intensity, personal care, lifting, sleeping, driving, recreation, headache, concentration, reading, and work. The items are scored from 0 (no limitations) to 5 (major limitations) and summed to create a total score reflecting degree of disability [24,29]: 0–4 = none; 5–14 = mild; 15–24 = moderate; 25–34 = severe; and over 34 = complete [29].

#### Range of motion

Range of motion (ROM) of the neck was measured in two-degree increments with a cervical measurement system [30]. The measurement system consists of a plastic helmet fitted with two spirit levels to judge and control the position of the head, two gravity goniometers and a compass to measure flexion, extension, lateral flexion, and rotation. The subject was seated in a chair with their back on a low back support, their head and shoulders in a neutral position, their hands on their thighs, and their feet on the floor. The subject was then asked to perform a full movement once in each direction. During the test, the test leader did not give any encouragement. Between each movement, the subject’s head was in the neutral position.

#### Neck strength

Maximal isometric neck strength was measured in neck flexion and neck extension by a handheld dynamometer (MicroFet 2, Hoggan), which is a stable and reliable device for measuring strength [31,32]. The flexion strength was measured with the subject in a supine position with legs straight and arms alongside the body. The upper cervical spine was flexed with the chin kept as close as possible to the chest. The extension strength was measured with the subject lying in a prone position and the head lifted and bent back as much as possible. The test leader gradually increased pressure on the forehead and the back of the head until the force was broken. During the test, the test leader did not give any encouragement. Each test was repeated three times and a mean value was calculated.

#### Shoulder strength

Strength of the shoulders was assessed by counting the number of two dynamic movements – arm abduction and upright row – with a pair of 4-kg dumbbells. The subject was told to do as many repetitions as possible, up to 50. When the subject did not manage to perform the whole movement, the test was stopped. It was easy for the physiotherapist to decide when the whole movement was not performed. During the test, the test leader did not give any encouragement. This way of measuring shoulder strength, rather than testing MVC, was chosen because the subjects’ chronic pain probably limited their ability of performing a maximal and immediate contraction in the affected area.

### Sample size

When estimating the sample size for analysing changes in pain intensity (one of the primary outcomes) *within the groups*, we assumed that the mean difference should have a standard deviation of 3. Expectation of a mean improvement of two points on the NRS, which also represents a clinically relevant improvement [33,34], required a sample size of 20 pairs of subjects to reject the null hypothesis with a power of 0.80 and a probability of <0.05 (two tailed).

When estimating the sample size for analysing changes in pain intensity (one of the primary outcomes) *between the groups*, we assumed that the mean difference should have a standard deviation of 3. Expectation of a mean improvement of two points on the NRS, which also represents a clinically relevant improvement [33,34], required a sample size of 36 subjects in each group to reject the null hypothesis with a power of 0.80 and a probability of <0.05 (two tailed). Sample size calculations were made using the computer program Power and Sample Size Calculations (v. 3.0.43, http://biostat.mc.vanderbilt.edu/wiki/Main/PowerSampleSize).

Based on the sample size estimations, also considering a probable presence of non-completers, and our available resources for running this trial, we aimed to include 50 subjects in each group.

### Completers and responders

Adherence to prescribed exercise was one main interest in this trial. Hence, completers and responders were defined as subgroups for result analyses.

#### Completer and non-completer

The exercise modalities included in our definition of a completer were neck and shoulder strength training for STRENGTH and stretching for STRETCH. In our definition, a completer reported at least eight unbroken weeks of exercise with a frequency of at least 1.5 times per week preceding the follow-up measurements (i.e., 4 to 6 months and 12 months). A non-completer was a subject that remained as a participant in the trial (i.e. still was exercising), but failed to reach the defined frequency of exercise per week valid for a completer. Data from the exercise diaries were used for the completer analysis.

#### Responder and non-responder

The responder definition for pain and function was based on criteria for clinically important changes in the two outcome areas. Thus, for neck pain and shoulder pain, a decrease of at least two points on the NRS was required [33,34]; for function, a decrease of the total NDI score of at least four points was required [35]. A non-responder was a subject that remained as a participant in the trial (i.e. still was exercising), but did not reach the defined level of improvement valid for a responder.

### Analyses and statistics

The results were analysed in two steps. First, the results within and between the allocated intervention groups were analysed according to both primary and secondary outcomes. Second, adherence to the exercise protocols were examined by analysing completers and non-completers to the prescribed interventions. In addition to the second step, analyses of how many of the participants who showed clinical improvement in primary outcomes were performed by analysing responders and non-responders.

To describe the changes within the groups, between the groups, and training frequency, median, the 25 and 75 percentiles, mean and standard deviation are given. For group comparisons, Mann Whitney U-test, Wilcoxon signed-rank test, and *X*^*2*^ tests were used. Comparisons were analysed using the baseline values as references (e.g., 4 to 6 months vs. baseline and 12 months vs. baseline). All statistics were performed using the statistical package IBM SPSS Statistics (version 19.0). For all tests, a probability of <0.05 (two-tailed) was accepted as the criteria for statistical discernibility. Participants who stopped exercising during the study period were excluded from the study and were not a part of the subsequent follow-up analyses.

## Results

The design of the comprised 50 subjects in each group. We managed to include 34 subjects in STRENGT and 23 subjects in STRETCH. This was due to a lack of eligible subjects available during a reasonable time-period for the recruitment. After inclusion, each subject was invited to three instruction sessions in order to learn the prescribed exercises. Most participants attended at least two instruction sessions.

Figure 1 shows the number of participants throughout the 12 months of exercising. During the 12-months of training, 12 STRENGTH participants and four STRETCH participants dropped out.

### Pain intensity, neck function, range of motion, and strength

The two intervention groups were similar with respect to age, pain intensities, pain duration, and function (NDI) at baseline (Table 1). Reported baseline values and outcomes at 4 – to 6 months and 12 months follow-ups for the two interventions groups are shown in Table 2.

**Table 2:** Within the groups: pain intensity (Numeric rating scale, NRS), function measured by Neck disability index (NDI), range of motion in the neck, and strength in the neck and shoulders at baseline after 4 to 6 months of training and 12 months of training

	**STRENGTH training group**				**STRETCHING group**					
	**Baseline**	**4 to 6 months**	**n**	**12 months**	**n**	**Baseline**	**4 to 6 months**	**n**	**12 months**	**n**
	**Median (25, 75 percentile)**	**Median (25, 75 percentile)**		**Median (25, 75 percentile)**		**Median (25, 75 percentile)**	**Median (25, 75 percentile)**		**Median (25, 75 percentile)**	
				***Primary outcomes***						
				*Pain intensity in the neck and shoulders*						
**Pain neck (NRS)**	6 (4, 7)	5 (2, 7)	25	2.5 (0, 7)	20	5 (4, 6)	4 (3, 8)	19	3 (2, 6)	19
**Pain shoulder (NRS)**	4 (3, 6)	4 (1, 7)	25	2.5 (0, 7)	20	5 (3, 8)	3 (0, 5)	19	3 (1, 5)	19
				*Function*						
**Function (NDI)**	13 (10, 18)	11 (7, 14)	25	10 (4, 12)	20	13.5 (11, 18)	11.5 (9.5, 15)	18	9 (4.5, 11.5)	17
				***Secondary outcomes***						
				*Range of motion in the neck (degrees)*						
**Neck flexion**	48 (38, 53)	54 (40, 60)	26	50 (47, 60)	22	48 (38, 50)	50 (42, 52)	19	48 (38, 62)	19
**Neck extension**	56 (46, 68)	70 (52, 79)	26	70 (63, 75)	22	60 (50, 68)	68 (56, 80)	19	70 (50, 78)	19
**Lat. flex. right**	30 (28, 38)	40 (36, 48)	26	40 (35, 42)	22	32 (22, 40)	38 (32, 42)	19	40 (34, 48)	19
**Lat. flex. left**	32 (30, 38)	39 (32, 43)	26	40 (37, 47)	22	38 (28, 40)	40 (32, 48)	19	42 (36, 52)	19
**Rotation right**	62 (57, 66)	69 (60, 75)	26	66 (60, 70)	22	64 (58, 68)	64 (54, 74)	19	68 (60, 70)	19
**Rotation left**	59 (52, 65)	68 (60, 73)	26	69 (60, 74)	22	62 (52, 72)	66 (58, 74)	19	68 (62, 72)	19
				*Strength in neck and shoulders (N) = Newton, (#) = numbers of repetition*						
**Neck flex, (N)**	56 (46, 63)	76 (68, 81)	26	75 (67, 82)	22	63 (55, 72)	75 (60, 85)	19	70 (59, 80)	19
**Neck ext, (N)**	92 (76, 110)	132 (118, 154)	26	123 (111, 149)	22	100 (74, 110)	121 (109, 146)	19	121 (102, 132)	19
**Shod. abd. 4 kg, (#)**	9 (5, 12)	15 (12, 18)	25	20 (14, 22)	22	9 (3, 13)	10 (7, 13)	16	11 (6, 12)	16
**Stand. row 4 kg, (#)**	18 (13, 25)	34 (26, 49)	26	38 (29, 50)	22	16 (14, 20)	20 (16, 30)	17	26 (20, 30)	17

### 4- to 6-month follow-up compared to baseline

#### Within group changes

STRENGTH reported a significant improved function (NDI). STRENGTH showed an overall increase of neck ROM, whereas STRETCH only improved neck extension and lateral flexion to the right. Both groups showed significant increases in neck and shoulder strength (Table 3).

**Table 3:** Within the groups: differences in pain intensity (Numeric rating scale; NRS), function measured by Neck disability index (NDI), range of motion in the neck, and strength in the neck and shoulders

	**STRENGTH training group**				**STRETCHING group**			
	**Difference BL - 4 to 6 months**	**p-value**	**Difference BL - 12 months**	**p-value**	**Difference BL - 4 to 6 months**	**p-value**	**Difference BL - 12 months**	**p-value**
	**Median (25, 75 percentile)**		**Median (25, 75 percentile)**		**Median (25, 75 percentile)**		**Median (25, 75 percentile)**	
			***Primary outcomes***					
			*Pain intensity in the neck and shoulders*					
**Pain neck (NRS)**	1 (−0.75, 3)	NS	2 (−1, 5)	NS	1 (−2, 2)	NS	1 (0, 2)	0.009
**Pain shoulder (NRS)**	1 (−2.75, 2.75)	NS	1 (−1, 3)	NS	1 (−1, 4)	NS	1 (0, 2)	0.017
			*Function*					
**Function (NDI)**	2 (0, 5.5)	0.036	4 (2, 8)	0.002	1 (−2, 4)	NS	4 (−1, 10)	0.015
			Secondary outcomes*					
			*Range of motion in the neck (degrees)*					
**Neck flexion**	−4 (−12.25, 2)	0.043	−2 (−9, 3)	NS	−2 (−12, 0)	NS	−6 (−16, 4)	NS
**Neck extension**	−9 (−15, -1.5)	0.004	−11 (−25, -2)	0.002	−6 (−19, -2)	0.002	−12 (−14, 0)	0.003
**Lat. flex. right**	−8 (−12, -2)	<0.001	−6 (−12, -2)	<0.001	−6 (−10, -2)	0.003	−6 (−14, 4)	<0.001
**Lat. flex. left**	−3.5 (−8.5, 0)	0.002	−8 (−10, 0)	0.002	−2 (−12, 4)	NS	−10 (−16, -2)	0.001
**Rotation right**	−6 (−10, 1)	0.002	−2 (−8, 1)	0.006	−2 (−10, 4)	NS	−4 (−14, 0)	NS
**Rotation left**	−9 (−12, -4)	<0.001	−10 (−13, -2)	<0.001	0 (−8, 4)	NS	−6 (−10, 2)	0.026
			*Strength in neck and shoulders (N) = Newton, (#) = numbers of repetition*					
**Neck flex, (N)**	−17 (−29, -9)	<0.001	−17 (−29, -12)	<0.001	−11 (−20, -1)	0.011	−5 (−22, 3)	NS
**Neck ext, (N)**	−30 (−55, -16)	<0.001	−40 (−57, -17)	<0.001	−28 (−44, -15)	0.002	−25 (−30, -12)	0.001
**Shod. abd. 4 kg, (#)**	−5 (−10, -1)	0.001	−8 (−16, -4)	<0.001	−1 (−4, 0)	0.044	−2 (−6, 0)	NS
**Stand. row4 kg, (#)**	−13 (−23, -5)	<0.001	−18 (−25, -6)	<0.001	−5 (−10, 0)	0.001	−6 (−13, -4)	0.003

BL compared to 4 to 6 months of training and BL compared to 12 months of training. Statistical analysis is performed with Wilcoxon signed rank test.

*An improvement in secondary outcomes is shown by a negative number (contrary to primary outcomes).

#### Differences between groups

No differences between the two groups were found in changes in the primary outcome pain intensities (p = 0.59-0.93) and function (NDI) (p = 0.50) at the 4–6 month follow-up.

Significant increases were found in favour of STRENGTH for the following secondary outcome variables: shoulder abductions ((median) STRENGTH: 4.0 vs. STRETCH: 1.0; p = 0.04) and standing row ((median) STRENGTH: 12.5 vs. STRETCH: 5.0; p = 0.02). Hence, no significant differences existed between the two groups in changes of the following secondary outcomes: ROM variables (p = 0.38-0.99) except for ROM neck rotation left ((median) STRENGTH: 9.0 vs. STRETCH: 0.0; p = 0.01) and strength of the neck (p = 0.09-0.52).

### 12-month follow-up compared to baseline

#### Within group changes

STRETCH reported reduced pain intensities in neck and shoulder. Both groups improved their function according to the NDI. STRENGTH improved ROM in all directions except neck flexion and STRETCH improved ROM in all directions except neck flexion and rotation to the right. STRENGTH showed overall increases in neck and shoulder strength, whereas STRETCH improved strength in neck extension and standing row (Table 3).

#### Differences between groups

No differences between the two groups were found in changes in the primary outcomes pain intensities (p = 0.50-0.91) and function NDI (p = 0.71) at the 12-month follow-up.

Significant increases were found in favour of STRENGTH for the following secondary outcome variables: flexion strength of the neck ((median) STRENGTH: 17.1 vs. STRETCH: 5.1; p = 0.031); shoulder abductions ((median) STRENGTH: 8.0 vs. STRETCH: 2.0; p = 0.01); and standing row ((median) STRENGTH: 17.5 vs. STRETCH: 5.5; p ≤ 0.00). No differences between groups existed in changes in ROM variables (p: 0.15-0.65) or extension strength of the neck (p = 0.09).

### Adherence to prescribed exercise dosage

**Figure 2:**
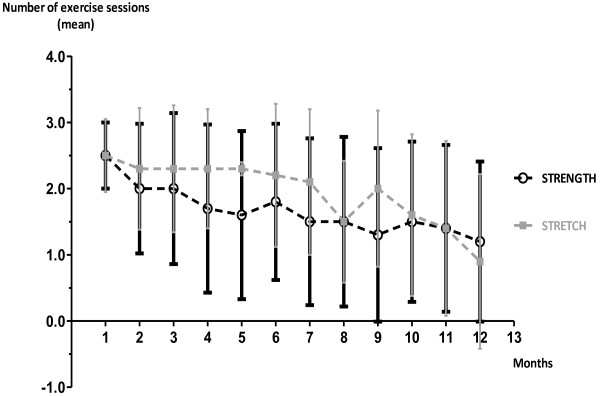
**Training frequency.** Training frequency during the trial showed in weekly mean frequency for each month for the strength training group (STRENGTH) and the stretching group (STRETCH).

There was a large variation in exercise frequency in both groups during the whole training period. From BL to the 4- to 6-month follow-up, the STRENGTH group performed exercises 1.5-2.5 times a week (Figure 2) and the STRETCH group performed exercises at least two times a week. After 4–6 months, the exercise frequency in STRENGTH was 1.5 times a week or less. STRETCH reported exercise frequency of 1.5 times a week or more until month 10. During the last two months of the trial, the STRETCH group performed the exercises less than 1.5 times a week.

### Completers and responders

Table 4 shows the number and proportions of completers and responders. Non-completers at 4–6 months had either dropped out or were still non-completers at 12 months.

**Table 4:** Number and proportions of completers, non-completers, and responders at 4 to 6-month and 12-month follow-up (n)

	**STRENGTH**	**STRENGTH**	**STRETCH**	**STRETCH**
	**4 to 6 months**	**12 months**	**4 to 6 months**	**12 months**
	**n = 24**^**a**^	**n =20**^**b**^	**n = 19**	**n = 19**
**Completers**	**19 (79%)**	**11 (55%)**	**17 (89%)**	**10 (53%)**
Responders neck pain	9 (47%)	5 (45%)	7 (41%)	4 (40%)
Responders shoulder pain	9 (47%)	6 (55%)	8 (47%)	5 (50%)
Responders function	7 (37%)	6 (55%)	5 (29%)	2 (20%)
**Non completers**	**5 (21%)**	**9 (45%)**	**2 (11%)**	**9 (47%)**
Responders neck pain	1 (20%)	5 (56%)	0	3 (33%)
Responders shoulder pain	1 (20%)	2 (22%)	1 (50%)	4 (44%)
Responders function	2 (40%)	5 (56%)	0	2 (22%)

^a^ = missing data from two subjects ^b^ = missing data from two subjects.

### 4- to 6-month follow-up for the completers and responders

#### Within group changes

At 4 to 6 months, there were 19 (79%) subjects that were completers and 5 (21%) non-completers in the STRENGTH group. Corresponding numbers for the STRETCH groups were 17 (89%) completers and 2 (11%) non-completers. In the STRENGTH completers, there were 9 (47%) responders for neck pain, 9 (47%) responders for shoulder pain, and 7 (37%) responders for function. The corresponding numbers for STRETCH were 7 (41%) responders for neck pain, 8 (47%) responders for shoulder pain, and 5 (29%) responders for function.

#### Differences between groups

At 4 to 6 months, there was no difference in proportions of completers between the groups (p = 0.28). Among the completers, there was no difference in proportions between the two intervention groups in responders for neck pain (p = 0.75), shoulder pain (p = 0.71), or function (p = 0.44).

### 12-month follow-up for the completers and responders

#### Within group changes

At 12 months, there were 11 (55%) subjects that were completers and 9 (45%) non-completers in the STRENGTH group. Corresponding numbers for the STRETCH group were 10 (53%) completers and 9 (47%) non-completers. In the STRENGTH completers, there were 5 (45%) responders for neck pain, 6 (55%) responders for shoulder pain, and 6 (55%) responders for function. The corresponding numbers for STRETCH were 4 (40%) responders for neck pain, 5 (50%) responders for shoulder pain, and 2 (20%) responders for function.

#### Differences between groups

At 12 months, there was no difference in proportions of completers between the groups (p = 0.90). Among the completers, there was no difference in proportions between the two intervention groups in responders for neck pain (p = 0.41), shoulder pain (p = 0.64), or function (p = 0.07).

### Harms

No important harms or unintended effects were found in either of the intervention groups.

## Discussion

Despite our very detailed planning and adequate financial resources, we did not manage to recruit a sufficient number of subjects within the available time. Hence, due to this lack of power, especially with respect to between group differences, the results have to be confirmed by larger studies and no definite conclusions with respect to the main aim can be drawn. In future studies, it may be important to consider other ways of recruiting subjects. With this pointed out we still believe it is important to discuss our results, as these results could be useful in the design of future studies.

A decrease in pain intensity and increase in function was found in the STRETCH group after 12 months of training. In the STRENGTH group, no pain decrease was reported but improved function was evident at both follow-ups. However, no differences between the groups were found at the two follow-ups for the primary outcomes – pain intensity and function.

Right from the start, adherence to the exercises was rather low and fell steadily, especially after six months of training. Adherence to prescribed activity and dosage reasonably influence outcomes of exercise [11], and evaluation of the effects of exercise should consider this aspect. Therefore, we defined the completers in this trial based on previously reported results [36,37] and our best assumption on a minimum training dosage needed to attain physiological and neuromuscular alterations presumably related to decrease in muscle pain. Thus, a completer performed at least half of the recommended exercise dose designed for in this study. Furthermore, to evaluate the clinical significance of pain relief and improvement in function, we defined a responder based on previous validations of NRS [33,34] and NDI [35].

To our knowledge, this trial is the first to report results based on both completers and responders. In line with the analyses of all the participants (STRENGTH and STRETCH), the completers in both groups showed similar improvements in pain intensity and function at the follow-ups. This finding contradicts what Ylinen [10], Andersen [8], and Zebis [9] found. These studies reported that specific neck strengthening exercises were superior to stretching [10], to general fitness training, to health promotion activities [8], and to advice about staying physically active [9].

There are, however, differences in study design that may explain some of the disparities in outcomes compared to our study. Unlike Ylinen et al. (2003), in our trial both STRENGTH and STRETCH received the same quantity and type of attention throughout the training period. Furthermore, in our trial no multimodal rehabilitation or manual treatments preceded the strength training. Rather few subjects comprised our trial as pointed out above, and concerning completers and responders the number of participants was even lower. However, clear results in favour of strength training despite few participants have been reported [8]. Andersen et al. provided supervised training, a protocol that probably improved exercise adherence [20]. In addition, the supervised training probably controlled and encouraged the performance of the prescribed exercises. Regular supervision was also one component in a large study examining the effectiveness of strength training [9] in which the participants performed the training at their workplace and during ordinary work hours (i.e., the design eliminated some common reasons why people do not exercise [21,38]).

There are, however, studies that do not show that strength training is superior to other types of exercise for improving pain and function [12-19]. Our result, based on the completer and responder analysis, that no convincing difference exists between high-intensity strength training and low-intensity exercise with respect to pain intensity and function agrees with the findings in other studies [16-19]. Presently, no obvious single factors in study design or exercise techniques explain the differences in outcomes [8-10,12-19]. In addition, one of the key conclusions in the Cochrane review on exercises for neck disorders [11] is that it has not been possible to determine the relative benefit of different exercise techniques and dosages. Søgaard et al. [39] suggests that different training such as general fitness training and specific strength training probably affects different underlying mechanisms that are involved in the pain condition. A recent review on chronic low back pain highlighted that improvements in muscle function were not always linked to decreased pain intensities [40]. This suggests that a probable mechanism for positive effects on pain as a consequence of exercise was central alterations and not peripheral improvements in the muscles. Similar connection was found in our trial in the within groups analysis where significantly increased strength in the STRENGTH group was not linked to a significant decrease in pain intensity, but the in the STRETCH group significant improvements in both strength and pain intensity were reported.

In our trial, eight of the 18 non-completers reported clinical improvements at the 12-month follow-up. For all participants, duration of pain was long, and different treatments had been tried (no detailed data are accessible) with little effect on neck pain. Spontaneous improvements in these eight non-completers are not probable. Instead, the improvements might be related to exercise performance over a sufficient period (12 months) even though the subject did not fit into the distinct definition of a completer. Through regular support and the training diaries, we know that the participants did perform varying amounts of training (i.e., weeks with good adherence alternated with weeks with low adherence). In addition, during the support sessions, many participants reported higher physical activity levels in their daily life. Thus, a continuous but somewhat irregular training for a long time, in combination with an increase in activity level in daily life, might result in decreased pain intensity and increased function, possibly explaining, at least in part, the improvements in non-completers.

As already mentioned, adherence to the exercise was not in accordance with the recommended dose and decreased throughout the training period. Physical exercise is a core component in the management of chronic pain conditions [11], so good adherence to prescribed exercise therapy is very likely to be essential. Forethought, planning, and rational decision making are common assumptions in theories that address adherence to health behaviour [41]. These assumptions are supposed to be preconditions for adherence and thus influenced the design and use of the diaries in this study. Furthermore, graded activity, individual focus, and simple strategies that address adherence (e.g., reminders, feedback, and support) have been shown to improve adherence [20]. The above-mentioned components were present and explicit in the home exercise concept. In spite of this, there is a considerable lack of adherence to prescribed exercises in this study. Lack of time, low motivation, and economic factors have been reported as reasons for non-adherence to prescribed physical activities [21]. The influence of these aspects is not assessed in our trial, but during the support sessions the participants often mentioned a lack of time as a reason for their inconsistent adherence. Presently, little is known about how inconsistent adherence to exercise interventions influences chronic pain [21].

### Limitations of the trial

Lack of power in this trial decreases the possibility of capturing statistically discernible differences *between* the groups. We aimed at including 50 participants in each group, but did not succeed due to practical reasons. Despite the lack of power, this trial provides interesting knowledge about statistically significant changes within the groups and a description of proportions of subjects reporting clinically relevant improvements in pain intensity and function. Neck function was fairly good when participants began the trial, a fact that presumably affects further improvement. This trial was only partially blinded, which could imply a potential bias. We were aware of this and tried to compensate for this methodological weakness with strictly structured measurement procedures where the test leader did not encourage the subjects’ performance during the tests. In addition, the two interventions were instructed and supported with the same structure and frequency to ensure an equal approach for all participants.

## Conclusions

We found no differences in the two primary outcomes between the two interventions, a finding that could be due to lack of statistical power. Both interventions based on home exercise resulted in improvements in pain intensity and function, which may implicate economic advantages compared to supervised interventions. However, home exercise requires a substantial motivation to ensure consistent adherence. It seems important, based on the relatively low adherence in the present study, that future research should include analysis of how to improve adherence for unsupervised exercise in subjects with chronic pain. In order to design clinically relevant studies, it may be important to focus both on power calculations and on estimations of adherence in order to achieve conclusive results. It is also essential to continue to examine exercise physiology and the relationships with pain physiology in order to optimize exercise therapies. A trial, even though under-powered as is the present trial, can give important clues concerning such mechanisms by analysing clinical responders both in completers and non-completers.

## Competing interests

The authors declare that they have no competing interests.

## Authors’ contributions

LK participated in the design of the study, implemented the interventions, and drafted the manuscript. EPT participated in the design of the study and drafted the manuscript. BG conceived of the study, participated in the design of the study, and performed some of the statistical analysis. BL conceived of the study, participated in the design of the study, participated in some of in the statistical analysis, and drafted the manuscript. All authors participated in the revisions of different versions of the manuscript and approved the final manuscript.

## Pre-publication history

The pre-publication history for this paper can be accessed here:

http://www.biomedcentral.com/1471-2474/15/6/prepub
